# Massive scrotal and abdominal subcutaneous emphysema secondary to intrascrotal heroin injection: A case report

**DOI:** 10.1016/j.eucr.2025.103115

**Published:** 2025-06-30

**Authors:** Narjes Saberi, Farid Rajaei Rizi, Mahmoud Valamehr

**Affiliations:** a Department of Urology, Isfahan Kidney Disease Research Center, Isfahan University of Medical Sciences, Isfahan, Iran; b Department of Urology, Al-Zahra Hospital, Isfahan University of Medical Sciences, Isfahan, Iran

## Abstract

This case report presents a rare instance of massive scrotal and abdominal subcutaneous emphysema resulting from self-injection of heroin. A 50-year-old incarcerated male exhibited painless scrotal swelling with extensive crepitus but no signs of infection or systemic toxicity. Imaging confirmed subcutaneous gas accumulation without visceral perforation or necrotizing fasciitis. Surgical exploration ruled out purulence or tissue necrosis, and the patient later admitted to intrascrotal heroin injections. The discussion underscores the importance of considering self-induced causes in diagnosing scrotal emphysema, particularly in high-risk populations. Early imaging, surgical evaluation, and psychiatric involvement are essential for optimal management.

## 1 Introduction

Pneumoscrotum, defined as the presence of air within the scrotal sac, is a rare clinical finding that can result from various etiologies, including trauma, infection, or iatrogenic causes.^1^ .Although most cases of pneumoscrotum are benign and self-limiting, its presence can occasionally indicate life-threatening conditions such as Fournier's gangrene or tension pneumothorax, necessitating urgent evaluation.^2^

Gas may enter the scrotal sac through various anatomical pathways, including direct scrotal entry, retroperitoneal or intra-abdominal dissection through the processus vaginalis, or migration from thoracic structures via fascial planes such as Scarpa's, Colles', and Dartos fascia.^3^

Despite the growing number of reported cases, instances of self-inflicted or idiopathic pneumoscrotum remain extremely rare and underrepresented in the literature.^3^

This report presents a uniquely rare case of pneumoscrotum resulting from self-inflicted scrotal air insufflation, in the absence of traumatic, infectious, or iatrogenic origin, where inconclusive imaging and diagnostic ambiguity necessitated surgical exploration.

Due to the extreme rarity and diagnostic uncertainty of non-infectious, idiopathic pneumoscrotum—particularly in cases requiring surgical exploration—this case adds valuable insights for clinicians facing similar unexplained presentations.

## 2 Case presentation

A 50-year-old incarcerated male was referred from a prison to the emergency department of Al-Zahra Hospital (Isfahan, Iran) in May 2025, presenting with acute scrotal swelling. He attributed the onset of symptoms to trauma sustained while getting out of bed. Initial history was obtained solely from the patient and revealed no known past medical conditions or comorbidities. He denied any history of substance use at presentation. On admission, he was alert and oriented, with stable vital signs: body temperature 37.3 °C, blood pressure 123/76 mmHg, heart rate 82 bpm, respiratory rate 16 breaths per minute, and oxygen saturation 98 % on room air. His body mass index (BMI) was calculated at 25 kg/m^2^. Physical examination revealed significant scrotal swelling with mild tenderness. Marked crepitus was palpable, extending superiorly to the lower abdominal wall. Due to the extent of edema, the testicular tissue could not be distinctly palpated. No skin ulceration, discoloration, or necrosis was observed. There were no systemic signs of infection such as fever, chills, or leukocytosis at that time.

Scrotal ultrasonography performed in the radiology department revealed extensive subcutaneous emphysema throughout the scrotal wall and extending into the inguinal canal, characterized by dirty shadowing and reverberation artifacts. No hematoma, hydrocele, torsion, or mass was detected. Due to the significant amount of air, testicular parenchyma could not be adequately visualized.

Laboratory studies revealed normal leukocyte count (WBC: 5700/mm^3^ with 73 % neutrophils), hemoglobin 13.4 g/dL, platelets 135,000/mm^3^, BUN 28 mg/dL, creatinine 1.2 mg/dL, and potassium 5.8 mEq/L. Coagulation profile and blood glucose were within normal limits.

**Fig. 1:**
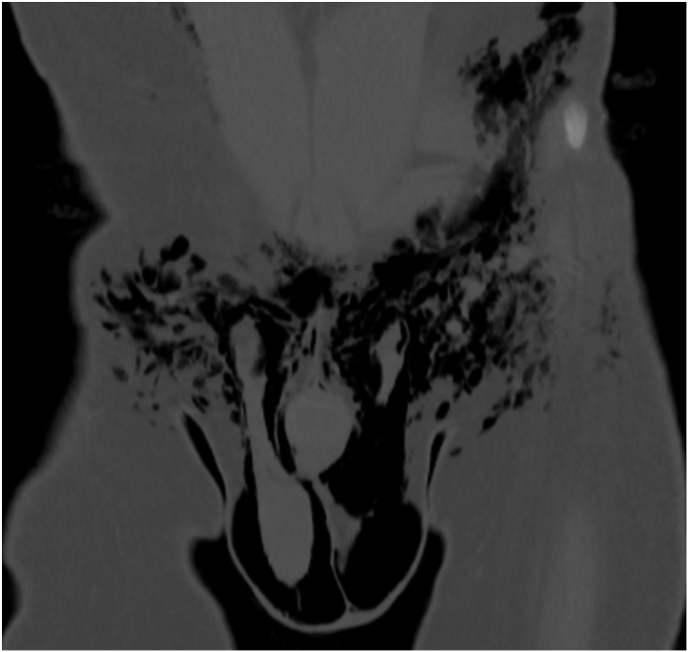
Non-contrast-enhanced computed tomography (CT) scan of the abdomen and pelvis demonstrating extensive subcutaneous emphysema localized to the superficial fascial planes of the scrotum and lower anterior abdominal wall.

A non-contrast-enhanced computed tomography (CT) scan of the abdomen and pelvis demonstrated extensive subcutaneous emphysema involving the scrotum, and lower anterior abdominal wall. No intra-abdominal free air, intra-luminal perforation, or evidence of bowel compromise was noted. The gas appeared localized to the superficial fascial planes without any deep fascial involvement or intramuscular gas extension (Fig. 1).

A plain chest X-ray revealed no evidence of pneumothorax or pneumomediastinum. Lung fields were clear, and the cardiac and mediastinal contours appeared within normal limits.

## 3 Operation

The patient underwent urgent surgical exploration under general anesthesia. A midline scrotal incision was performed by the urology team, followed by a Pfannenstiel incision by general surgery due to the extension of subcutaneous emphysema to the lower abdominal wall.

**Fig. 2:**
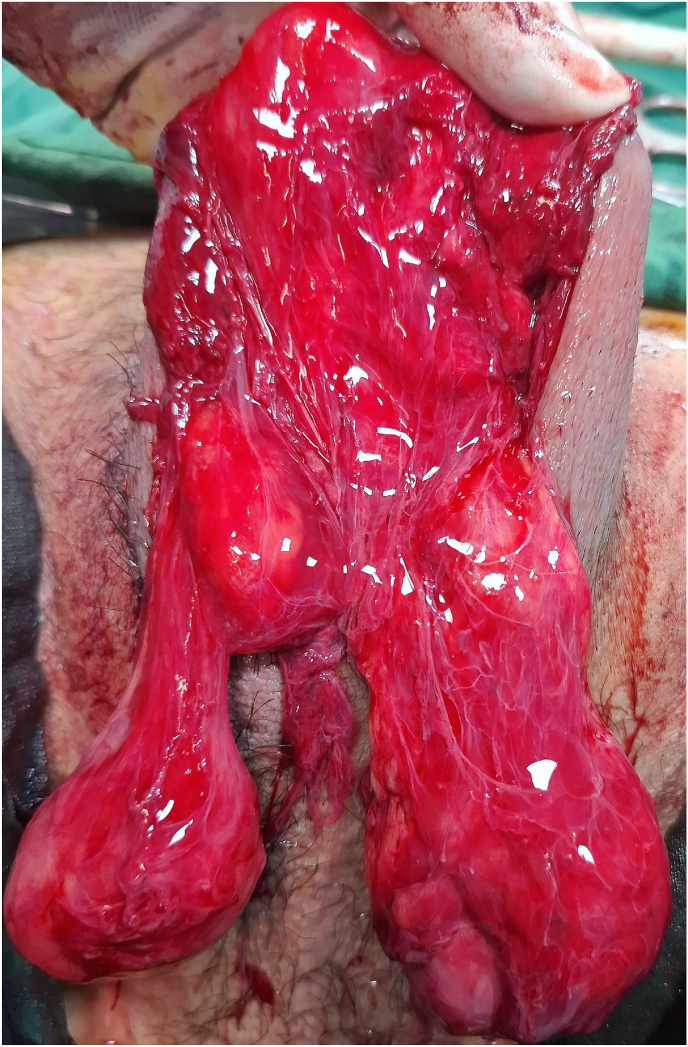
No purulent discharge, tissue necrosis, abscess formation, or evidence of Fournier's gangrene was observed. Both testes were intact and viable despite being non-palpable due to severe edema.

Intraoperatively, no purulent discharge or necrotic tissue was observed. Both testes were intact and viable but were initially non-palpable during physical examination under anesthesia due to severe edema. The subcutaneous tissue contained a significant amount of gas, producing a characteristic crackling sensation upon palpation, similar to bubble wrap. No signs of abscess, tissue necrosis, or Fournier's gangrene were detected (Fig. 2).

No drains were inserted. The surgical wounds were left open and managed with daily irrigation and dressing changes. After confirming the absence of infection and observing healthy granulation tissue, delayed primary closure was performed approximately four days later.

Empirical broad-spectrum intravenous antibiotics (meropenem and clindamycin) were initiated upon admission and continued postoperatively. On the second postoperative day, the patient admitted to intentionally injecting heroin into his scrotum—a fact he had initially denied. A psychiatric consultation was conducted, and the patient was referred for further addiction management and mental health support.

## 4 Follow-up and outcome

The patient's postoperative course was uneventful. Daily wound care and irrigation were performed under sterile conditions. No signs of infection, necrosis, or wound dehiscence were observed.

On the fourth postoperative day, as the local inflammation subsided and healthy granulation tissue developed, the wound was closed primarily without complications.

The patient was discharged in a stable condition on postoperative day 8 with complete resolution of scrotal swelling and no clinical signs of systemic or local infection.

He was followed up in the outpatient urology clinic at one week and one month after discharge. The surgical wounds had healed completely, and there were no residual symptoms or complications. Scrotal ultrasound at one-month follow-up revealed normal echotexture and perfusion of both testes.

Psychiatric follow-up confirmed a diagnosis of opioid use disorder. The patient was referred to a structured addiction treatment program, including behavioral therapy and pharmacologic support. He reported abstinence from injection drug use at his one-month visit and expressed willingness to continue psychiatric care.

## 5 Discussion

This case highlights the diagnostic dilemma posed by atypical presentations of scrotal emphysema in patients without overt signs of infection, particularly when accompanied by unreliable or unclear patient histories. In such presentations, Fournier's gangrene—a rapidly progressive necrotizing fasciitis—must be ruled out promptly. However, rare non-infectious etiologies such as spontaneous subcutaneous emphysema should also be considered in the differential diagnosis.^4^^,^^5^

Although CT imaging did not confirm the origin or route of the subcutaneous emphysema, it was useful in assessing the extent of tissue involvement. Given the persistent clinical suspicion of necrotizing soft tissue infection, surgical exploration was undertaken without delay, as recommended by current guidelines.

The patient's initial denial of substance use, coupled with the absence of overt signs of infection, made it difficult to distinguish between Fournier's gangrene and other non-infectious causes of scrotal and abdominal subcutaneous emphysema.

Considering the extensive subcutaneous emphysema and the discordance of this case with previous self-inflicted presentations by incarcerated individuals—who typically injected air into the scrotum—a decision was made to proceed with surgical exploration. Intraoperatively, the volume and distribution of subcutaneous air appeared inconsistent with patient-administered injection. However, in the absence of an identifiable internal source and following postoperative confession by the patient, a diagnosis of self-inflicted subcutaneous emphysema was ultimately established.

What made this case particularly unique was the unusually extensive spread of subcutaneous emphysema, which significantly distinguished it from other self-inflicted cases previously encountered at our center. In prior instances—most of which occurred among incarcerated individuals—air had typically been injected using a needle, resulting in limited and superficial emphysema that was easily drained without the need for surgical exploration. However, the magnitude of emphysema in this patient initially mimicked a life-threatening necrotizing infection, thereby complicating the diagnostic process. It is well-documented that inmates may resort to such self-inflicted injuries to escape the prison environment, motivated by reasons such as requesting medical leave, inability to tolerate confinement, or even attempts to escape. This case stands out as a rare and deceptive presentation within this context.Managing such cases requires not only clinical discernment but also ethical and legal sensitivity, as these patients may deliberately induce symptoms to obtain temporary release, medical leave, or other secondary gains.

"Although rare, self-inflicted subcutaneous emphysema has been documented in incarcerated individuals seeking secondary gains, such as temporary release or improved living conditions. Previous case reports have described similar presentations in prisoners, including facial emphysema and pneumomediastinum, used as a means of simulating medical emergencies to facilitate hospital transfer.^6^^,^^7^

## 6 Conclusion

This case highlights the importance of maintaining a high index of suspicion for necrotizing soft tissue infections when confronted with extensive subcutaneous emphysema, particularly in high-risk populations such as incarcerated individuals. Although rare, self-inflicted subcutaneous emphysema can clinically and radiologically mimic life-threatening infections, warranting urgent evaluation and sometimes surgical exploration. Awareness of factitious disorders and their psychosocial motivations is crucial to avoid unnecessary interventions, while simultaneously ensuring that true surgical emergencies are not missed.

## CRediT authorship contribution statement

**Narjes Saberi:** Methodology, Supervision, Visualization, Writing – original draft, Writing – review & editing. **Farid Rajaei Rizi:** Conceptualization, Data curation, Formal analysis, Investigation, Methodology, Project administration, Resources, Software, Supervision, Validation, Visualization, Writing – original draft, Writing – review & editing. **Mahmoud Valamehr:** Writing – original draft, Writing – review & editing.

## Ethical aspects

Written informed consent was obtained from the patient for the publication of this case report and accompanying images. The study was conducted in accordance with the 1964 Helsinki declaration and its later amendments.

## Use of AI assistance for language refinement

To enhance the clarity and readability of this case report, artificial intelligence (AI)-based language processing tools were utilized for paraphrasing and grammatical refinement. These AI tools assisted in restructuring sentences while preserving the original meaning, ensuring adherence to standard grammatical conventions and improving overall coherence. The integration of AI-driven editing facilitated the production of a more polished and scientifically precise manuscript while maintaining the integrity of the clinical content. All AI-generated modifications were carefully reviewed to confirm accuracy and appropriateness before final inclusion in the report.

## Funding

This research did not receive any specific grant from funding agencies in the public, commercial, or not-for-profit sectors.

## Conflict of interest

The authors declare that they have no conflict of interest.
